# Comparing the effects of non-homogenous mixing patterns on epidemiological outcomes in equine populations: A mathematical modelling study

**DOI:** 10.1038/s41598-019-40151-2

**Published:** 2019-03-01

**Authors:** Rachael M. Milwid, Terri L. O’Sullivan, Zvonimir Poljak, Marek Laskowski, Amy L. Greer

**Affiliations:** 1 Department of Population Medicine, University of Guelph, Guelph, ON Canada; 2 Department of Mathematics and Statistics, York University, Toronto, ON Canada

## Abstract

Disease transmission models often assume homogenous mixing. This assumption, however, has the potential to misrepresent the disease dynamics for populations in which contact patterns are non-random. A disease transmission model with an SEIR structure was used to compare the effect of weighted and unweighted empirical equine contact networks to weighted and unweighted theoretical networks generated using random mixing. Equine influenza was used as a case study. Incidence curves generated with the unweighted empirical networks were similar in epidemic duration (5–8 days) and peak incidence (30.8–46.4%). In contrast, the weighted empirical networks resulted in a more pronounced difference between the networks in terms of the epidemic duration (8–15 days) and the peak incidence (5–25%). The incidence curves for the empirical networks were bimodal, while the incidence curves for the theoretical networks were unimodal. The incorporation of vaccination and isolation in the model caused a decrease in the cumulative incidence for each network, however, this effect was only seen at high levels of vaccination and isolation for the complete network. This study highlights the importance of using empirical networks to describe contact patterns within populations that are unlikely to exhibit random mixing such as equine populations.

## Introduction

Disease transmission models are used to examine disease dynamics and the associated effects of implementing different intervention strategies in the population^1^. In the models, infectious individuals can infect susceptible individuals at a rate, *β*, which comprises the probability of infection and the contact rate^2^. Often, disease transmission models assume that the entire population has an equal probability of coming into contact^3^. This assumption is referred to as the assumption of homogenous mixing, and, if not representative of the actual contacts that occurred in the population, can result in incorrect model predictions^3^. One method of correcting for the assumption of homogenous mixing is to incorporate heterogeneous population mixing.

Heterogeneous contact patterns can be integrated into the model by defining contact networks explicitly^4^. Contact networks describe the rate and frequency of the contacts that occurred between individuals in the population of interest. The simulation of disease transmission over a contact network is referred to as a network epidemic model^2^. The nodes in network epidemic models represent individual entities such as people, places, or animals, while the edges between the nodes represent contacts between the individual nodes through which disease transmission can occur^5^. While empirical networks describing the effective contacts that occur within a population can be useful, it can be challenging to collect these data^6,7^. Different methods have been used to simplify the process of simulating disease dynamics on networks including the use of summary statistics (i.e. network centrality) to generate representative theoretical networks^5^. Common theoretical networks include random networks, small-world networks, and scale free networks^8^. Random networks are characterized by random edge formation between nodes. Random networks tend to have small path lengths and minimal clustering^7^. While small-world networks also have short path lengths, they are characterized by high clustering^9^. Lastly, scale free networks follow a power law distribution such that most nodes have few connections while a few nodes have many connections^7,10^. In particular, a random network in which each node is connected to every other node is referred to as a complete network. Complete networks are often used to represent the assumption of homogenous mixing^11^.

It has long been recognized that the different network structures can affect the disease dynamics that occur within a network^12^. This conclusion has been established through combined theoretical studies and modelling approaches^11–14^. Given the recent advances in technology, combined with the improved level of record keeping for animal populations, the collection of contact pattern and movement data has become more common. This type of data has been used in past studies to explicitly model the transmission of various pathogens through both human^15–18^ and animal^19–21^ populations. However, to date, most published studies that focus on disease transmission modelling of equine infectious diseases assume homogenous mixing which may not correctly characterize the types of contact that generally occur within equine populations. This is largely attributable to the scarcity of equine contact pattern data. Furthermore, given the paucity of published equine infectious disease data, studies tend to focus on Equine Influenza (EI), for which published natural history and incidence data are available. Equine influenza is a highly transmissible respiratory disease caused by the equine influenza virus^22^. The disease is most commonly spread by direct contact with an infectious horse, but transmission is also possible via indirect mechanisms such as: aerosols, wind, and fomites^22–24^. While EI has a morbidity rate of up to 100%^23^, it has a low mortality rate, and tends to be self limiting^25^.

Prevention and control strategies for equine influenza include vaccination of susceptible horses, quarantine of exposed horses, and/or isolation of infectious horses^26^. There are currently two types of vaccines available in Canada; a vaccine containing the killed virus and a vaccine containing a modified live virus^27^. Neither type of vaccine confers full protective immunity against infection^25^. Since the vaccine efficacy is highly dependent on an individual’s antibody levels against the glycoprotein haemagglutinin^23^, the efficacy is improved when the vaccine strain matches the challenge strain^23^. There are currently no regulatory requirements with respect to EI vaccination or biosecurity measures in Canada. It is, however, advised that susceptible horses get vaccinated, and that horses are quarantined for a two-week period prior to being introduced into a new facility^27^. Therefore, the population level immunity against EI is likely to differ based on the population of interest.

Although vaccination, quarantine, and isolation are recommended biosecurity practices for the prevention and containment of EI^22,28^, existing disease transmission models for EI have focused primarily on vaccination as an intervention. These studies have modeled the transmission of EI in both vaccinated and unvaccinated populations^29–34^. The results of the existing studies have highlighted the importance of vaccination as a method for reducing the EI disease burden in equine populations.

The purpose of this study was to assess the impact of using empirical contact network data to inform the contact rate of equine disease transmission models, as well as to assess the effect of using both vaccination and isolation as intervention strategies while using equine influenza as a case study. These research objectives were addressed through a multi-step process that included the collection, analysis, and utilization of both weighted and unweighted empirical contact pattern data to inform the development of network epidemic models. Briefly, empirical contact pattern data were collected using active radio-frequency identification (RFID) proximity sensing tags from 4 equine facilities in Canada. The data were used to generate both weighted and unweighted contact networks for each facility, each of which was used to inform the contact rate of a disease transmission model for equine influenza. In addition to the models that incorporated empirical contact networks, the model was also simulated using random mixing theoretical networks that were generated using the characteristics of the empirical networks. The results of the disease transmission model informed by the individual networks (both empirical and theoretical) were compared to assess the impact of the different network structures on the disease dynamics. Given the different network structures, the study hypothesis was that the model simulations would result in different outcomes and trends for each network examined.

## Results

### No-intervention model

#### Unweighted networks

**Figure 1:**
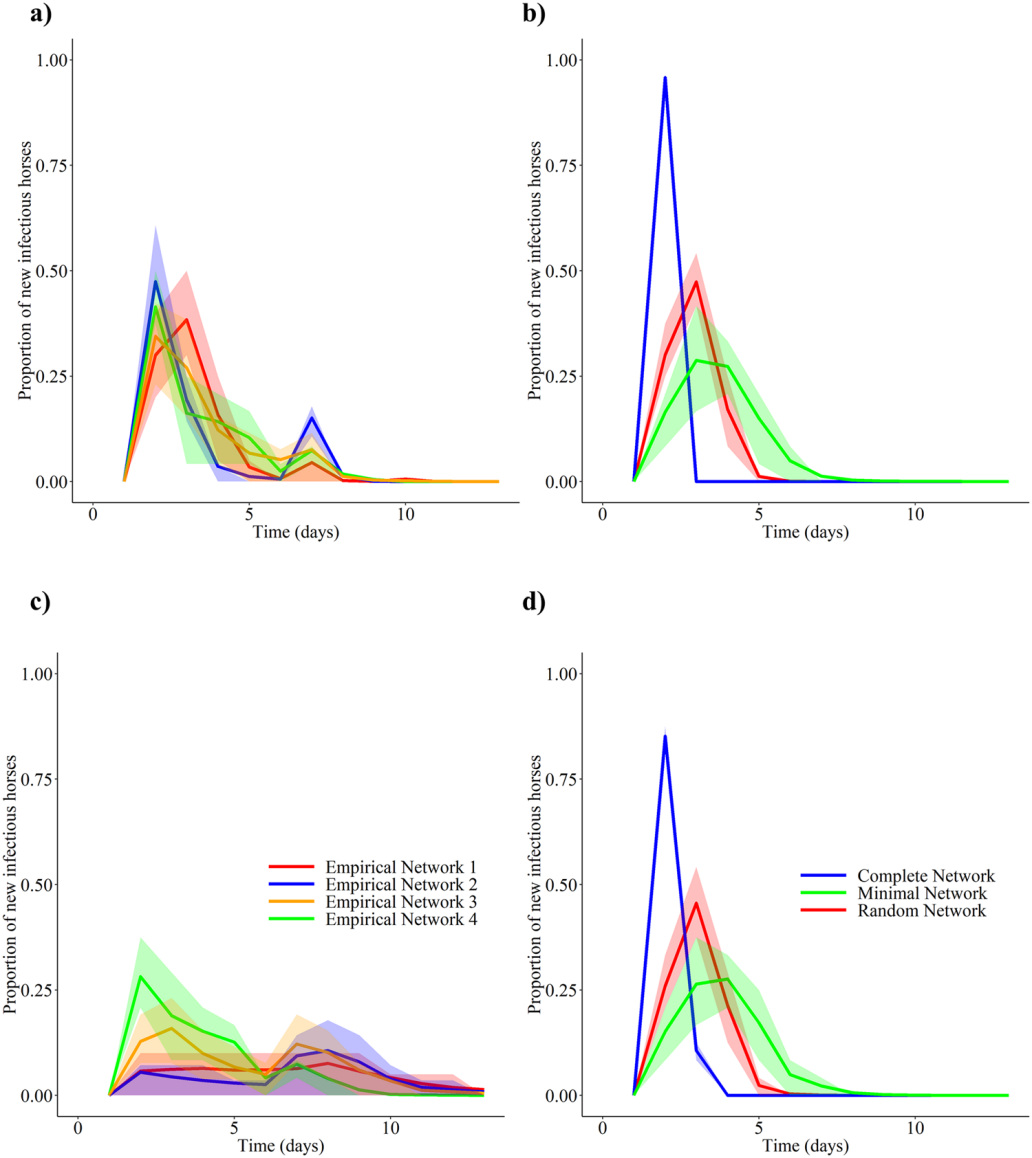
Epidemiological results for the disease transmission model in the absence of interventions. The bands represent the 25 and 75% quantiles. Panels (a,b) contain plots of the infection incidence over time when the contact rate was informed using unweighted empirical and theoretical networks respectively. Similar curves were produced for each empirical network and the Random Mixing Network. The peak incidence was the smallest for the Minimal Network, followed by the Empirical Networks and the Random Mixing Network. The peak incidence was the largest for the complete network. Panels (c,d) contain plots of the incidence over time when the contact rate was informed using weighted empirical and theoretical networks respectively. Each empirical network resulted in unique incidence curves. The theoretical networks resulted in similar curves to those produced using the unweighted networks.

The base model, in which no interventions were implemented, resulted in similar epidemic curves for each empirical network, as well as the random network, with respect to the epidemic duration, and the epidemic peak height and time (Fig. 1a-b and Table 1). While the Minimal Network had the smallest peak incidence (25%), the duration of the generation of new cases (7 days) was similar to the Random Mixing Network (RMN) and empirical networks (5 and 5–8 days). The peak incidence for the Minimal Network occurred on day 3 of the simulation. In contrast to the theoretical networks, the incidence curves for each empirical network were bimodal (Fig. 1). The first epidemic peak (30.8–46.4%) was always larger than the second peak. The peak incidence occurred at approximately the same time for each of the empirical networks (days 2–3). By days 5–8, the incidence had decreased to 0. The peak incidence for the RMN was slightly smaller (45.8%) than the peak incidence for the empirical networks and occurred on day 3 of the simulations. The incidence had decreased to 0 by day 5 of the simulation. The Complete Network had the largest peak incidence (95.8%) which occurred at approximately the same time as the other networks (day 2). The generation period of new EI cases was the shortest for the Complete Network (3 days).

**Table 1:** Key epidemiological results for the disease transmission model of equine influenza.

	Size of peak incidence		Time of epidemic peak (day)		Time until no new infections (days)	
	Weighted network (%)	Unweighted network (%)	Weighted network	Unweighted network	Weighted network	Unweighted networks
Empirical Network 1	5.0	35	5	3	10	5
Empirical Network 2	7.14	46.4	8	2	15	8
Empirical Network 3	15.39	30.8	3	2	10	8
Empirical Network 4	25.0	37.5	2	3	8	8
Random Mixing Network	41.7	45.8	3	3	5	5
Complete Network	83.3	95.8	2	2	4	3
Minimal Network	25.0	25.0	3	3	7	7

The base model had an SEIR structure and did not include any disease prevention or control interventions. The epidemic model was simulated on different base networks including 4 empirical networks (“Empirical Networks 1–4”), a network with the average characteristics (average degree, and network size) of the empirical networks (“Random Mixing Network”), a Complete Network in which all the nodes were connected to each other, and a network with an average degree of 4 (“Minimal Network”). The empirical networks and the Random Mixing Network had similar epidemiological characteristics. The Complete Network had the largest epidemic peak and the shortest epidemic duration. The Minimal Network had the smallest epidemic peak and the longest epidemic duration.

#### Weighted networks

Similar patterns were observed for the weighted theoretical networks, in which the edge weight represented the total contact duration between horses in each network (Fig. 1d and Table 1). In contrast to the unweighted Empirical Networks, the weighted Empirical Networks and the Minimal Network had the smallest peak incidence (~5–25%) followed by the RMN (~41.7%), and the Complete (~83.3%) networks. Furthermore, a larger difference was observed in the peak height of the incidence curves for each of the Empirical Networks when the weighted networks were used, as opposed to when the unweighted networks were used to inform the model. Lastly, the generation period of new EI cases was the longest for the Empirical Networks (8–15 days) compared to the Complete Network (4 days), RMN (5 days), and the Minimal Network (7 days).

### Vaccination and isolation model

#### Unweighted networks

**Figure 2:**
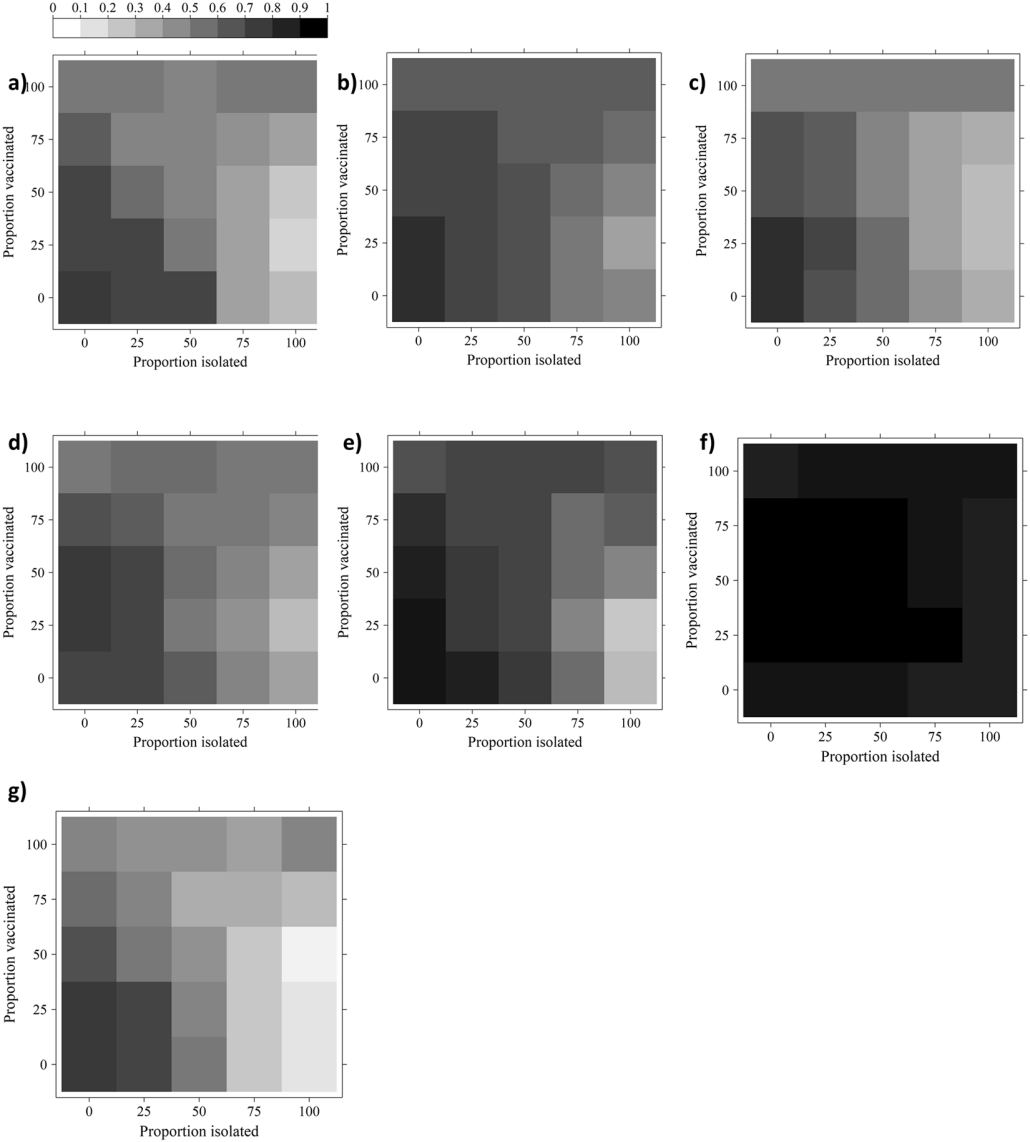
Heat maps of the cumulative incidence (%) when isolation and vaccination were implemented in the population. The heat maps represent unweighted Empirical Networks 1–4 (panels (a–d)), the unweighted Random Mixing Network (panel (e)), the unweighted Complete Network (panel (f)), and the unweighted Minimal Network (panel (g)). Increasing the proportion of the population isolated and/or vaccinated generally decreased the cumulative incidence. A non-linear trend in the cumulative incidence was observed for high isolation rates used in addition to a vaccination program.

Similar trends were observed in the heat maps describing the cumulative incidence, defined as the total number of new infectious cases divided by the number of nodes in the network, in all the networks other than the Complete Network (Fig. 2). In the majority of the networks, the implementation of an isolation-only strategy, in which infectious horses were isolated for a 14-day period, resulted in a distinct decrease in the cumulative incidence with an increase in the proportion of infectious horses isolated. However, an increase in the proportion of vaccinated horses in a vaccination-only strategy, had minimal effect on the cumulative incidence until vaccination rates of 50% had been reached. For the majority of the mixed vaccination-isolation intervention strategies, an increase in the isolation rate had a greater effect on reducing the cumulative incidence than an increase in the vaccination rate. The incorporation of any isolation strategy in addition to a 100% vaccination rate had minimal effect on the cumulative incidence and resulted in a cumulative incidence of 50–64%. In contrast, increasing the vaccination rate when high levels of isolation (~50–100%) were used caused a decrease in the cumulative incidence. This trend was evident until a ~25–50% vaccination rate had been achieved, after which the cumulative incidence increased again (Fig. 2a–d).

The trends in the cumulative incidence for the RMN and the Minimal Network were similar to those of the empirical networks (Fig. 2e,g). Furthermore, the non-linear effect of vaccination used in conjunction with an isolation program was less obvious in the Minimal Network than in the other networks. The implementation of a vaccination and/or isolation program decreased the cumulative incidence for both the RMN and the Minimal Network. In contrast, the implementation of any intervention program on the Complete Network was ineffectual (Fig. 2f).

#### Weighted networks

**Figure 3:**
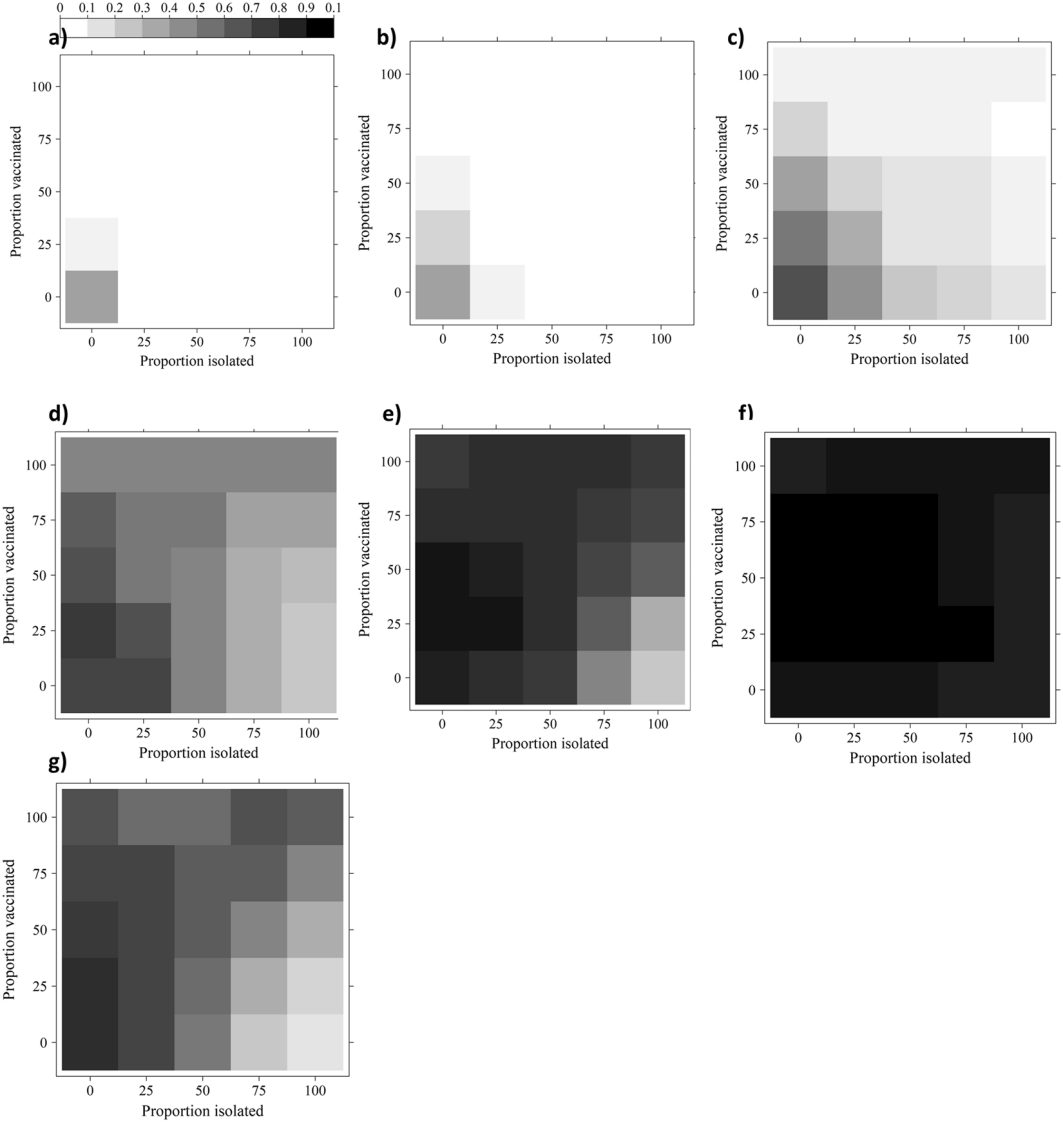
Heat maps of the cumulative incidence (%) resulting from different isolation and vaccination combinations implemented in a model whose contact rate was informed with weighted contact networks. The heat maps represent Empirical Networks 1–4 (panels a–d), the Random Mixing Network (panel e), the Complete Network (panel f), and the Minimal Network (panel g). Increasing the proportion of the population isolated and/or vaccinated generally decreased the cumulative incidence. While it was possible to decrease the cumulative incidence of Equine Influenza to 0–10% for Empirical Networks 1 and 2, it was unlikely for the remaining networks.

The incorporation of the weighted Empirical Networks in the model resulted in a wide range of disease dynamics (Fig. 3). In each of the Empirical Networks, the incorporation of any intervention strategy reduced the cumulative incidence of infection. However, for Empirical Networks 1 and 2, it was possible to reduce the incidence of EI to 0–10% with a single strategy intervention of at least 50% vaccination for Empirical Network 1, 75% vaccination for Empirical Network 2, or 25% isolation for both Empirical Networks 1 and 2 (Fig. 3a,b). In contrast, with the exception of a 100% isolation program combined with a 75% vaccination program for Empirical Network 3, it was impossible to reduce the EI incidence to levels of 0–10% for Empirical Networks 3 and 4 (Fig. 3c,d). For both Empirical Networks 3 and 4, a combined program of at least 25% isolation and 50% vaccination was required to reduce the cumulative incidence to 50%, or a singular program of high vaccination levels (>75%) or 50% isolation.

Each of the theoretical, random mixing networks resulted in different heat maps (Fig. 3e–g). The minimal network resulted in the smallest cumulative incidence, while the complete network resulted in the largest cumulative incidence, where every horse in the population became infected. In order to reduce the cumulative incidence to at most 50% in the minimal network, isolation levels of at least 25%, or vaccination levels of at least 50% were required (Fig. 3g). In contrast, high levels of isolation (75–100%) combined with low to medium levels of vaccination (0–50%) were required to reduce the cumulative incidence in the Random Mixing Network to 50% or less (Fig. 3e).

## Discussion

Limited data exists describing herd-level mixing patterns of agricultural animals^20^. This data deficiency is especially true for the equine population making it difficult to develop realistic disease transmission models. Therefore, it is not surprising that few mathematical modelling studies have focused on facility-wide transmission dynamics of equine pathogens including influenza. Furthermore, given the paucity of equine contact pattern data, the models that have focused on EI have tended to assume homogenous mixing within the population of interest. The current study used data collected with RFID tags to quantify the effects of heterogenous mixing patterns on the dynamics of disease spread.

The networks generated for this study were used to assess the effect of the following network structure characteristics on disease spread dynamics: (1) weighted vs. unweighted networks, (2) empirical vs. theoretical networks, and (3) network density and size. It was hypothesized that the different network structures would result in distinct epidemiological outcomes. While this hypothesis was incorrect with respect to the unweighted, empirical networks, it was correct with respect to the weighted networks. In contrast to the study hypothesis, the model simulations produced similar epidemiological outcomes for each unweighted empirical network. One explanation for this outcome is the law of mass action. The law of mass action states that the number of new infections when susceptible individuals (*S*) come in contact with infectious individuals (*I*) is proportional to *SI*^35^. The proportionality constant is often referred to as the transmission rate, *β*, and consists of the contact rate and the probability of transmission given an effective contact^36,37^. In each participating facility, groups of horses were turned out together, forming a subpopulation in which homogenous mixing could occur. During the turnout period, the horses’ halters and the attached RFID tags were stored together on the pasture fence, inferring that the horses were in contact for the duration of the turnout period. The horses remained in their pastures for the majority of the day, until they were returned to their stalls in the evening. The horses were removed from their pastures for training and pleasure riding, and during this time, could come in contact with horses from the other pastures. Therefore, it is likely that the law of mass action occurred on a smaller scale, within the pastures, and could be the reason for the similarity in the epidemic outcomes observed. Conversely, the incorporation of weighted edges in the networks resulted in a difference in the disease dynamics within the Empirical Networks. Although this difference could be attributed to the different transmission probability, $\tau$, used in the weighted and unweighted networks, a corresponding difference was not observed between the weighted and unweighted theoretical networks. Therefore, the difference in the disease dynamics can likely be attributed to the added dimension of heterogeneity between the networks with respect to the edge weights.

The structured degree distribution directly correlates with disease propagation due to its relationship with the transmission rate^38–40^. Since it has been shown that local structure slows disease spread when compared to random mixing^14^, the underlying structure in the degree distribution of the empirical networks can explain the discrepancy in the shape of the incidence curves between the theoretical and empirical networks. Additionally, the different network densities likely contributed to the different disease dynamics between the networks.

The implementation of a combined vaccination and isolation program in the model resulted in unexpected patterns in the cumulative incidence of the unweighted networks. The cumulative incidence generally decreased with an increase in the respective intervention strategy. However, higher isolation rates used in addition to a vaccination program lead to a non-linear trend in the cumulative incidence with increasing levels of vaccination. The non-linear trends in the cumulative incidence may be indicative of a backward bifurcation. A detailed description of a backward bifurcation can be found in^41^. Briefly, bifurcation analysis relates the stability of a system to the basic reproduction number, *R*_0_^41,42^. An ${R}_{0} < 1$ is generally representative of possible disease eradication, while an ${R}_{0} > 1$ implies disease persistence^42,43^. Consequently, a health program should endeavour to maximize the realistic intervention strategies in order to decrease ${R}_{0}$ to less than 1. However, a backward bifurcation implies that decreasing the ${R}_{0}$ to less than 1 will not necessarily eliminate a disease^41,42^. Known model structures can cause a backward bifurcation, including the incorporation of an imperfect vaccine, multiple groups such as multiple susceptible groups with different characteristics, and differential susceptibility to infection^41,42,44^. Given the trends in the cumulative incidence with respect to the vaccination rate it is possible that the model is portraying a backward bifurcation. Therefore, the model results indicated a specific level of vaccination which will minimize the cumulative incidence, however, a higher or lower vaccination rate will cause an increase in the infection incidence. Mechanistically, the non-linear relationship can be explained by the use of an imperfect vaccine. At high levels of vaccination, a large proportion of the population are likely to be asymptomatic shedders, inhibiting the ability to isolate infectious horses, and increasing disease transmission within the population. This trend however, was not observed in the weighted networks. Therefore, future work should focus on understanding the drivers of the non-linear relationship between high levels of isolation coupled with vaccination.

With respect to the unweighted theoretical networks, the cumulative incidence of the Minimal Network was the smallest, and the cumulative incidence of the Complete Network was the largest. The relative cumulative incidence for each of the theoretical networks can be attributed to the network degree and density which was smallest for the Minimal Network, and largest for the Complete Network. These results correspond to the study by May *et al*. (2001), in which the authors examined the effect of increasing the average degree with respect to the total number of individuals that became infected during the course of the epidemic. The authors concluded that an increase in the network connectivity corresponded to an increase in the number of individuals infected. Therefore, in networks with a high connectivity, all individuals became infected^14^.

The epidemiological results of this study were consistent with the results of Glass *et al*. (2002) in which the authors used a stochastic model to describe the effects of vaccination on the epidemiology of EI. Glass *et al*. (2002) concluded that vaccine uptake decreased the epidemic size. The model was validated with data obtained from a New York race track. The validation data indicated that the incidence curve was bimodal with new cases occurring for a duration of 1 month. The fitted model, however, was unimodal with the development of new cases for a duration of 20 days. The incidence curves from the current study were also bimodal (for the empirical networks), with new cases occurring for 5–8 days in the unweighted networks, and 8–15 days in the weighted networks. One possible explanation for the discrepancy in the duration of the generation of new cases is the stochastic nature of the model. The study results were averaged over 10,000 simulations, meaning, that some of the longer epidemic durations were likely consistent with the results from the Glass study. Furthermore, race facilities are managed differently than sport facilities, and these differences may contribute to the difference in observed outcomes. For example, in order to avoid injury, horses at a racing facility may be turned out individually as opposed to the group turnouts practiced by the participating equine facilities.

While the study results highlight the importance of using detailed, empirical data for populations such as the equine population, there are limitations. First, the network sizes were relatively small. In their study, Glass *et al*. (2002) concluded that the population size did not affect the model outcomes, however, this result might not hold for heterogenous mixing patterns. Second, the assumption that horses were in contact during turnout might have over-estimated the contact rates for the horses that shared a pasture. More detailed information regarding the duration of specific contacts between the horses that shared a pasture might result in different outcomes, as the number of horses that each horse contacted might decrease. Lastly, the complexity of incorporating time varying networks in the model made the application of certain analyses difficult, including the calculation of ${R}_{0}$, and a bifurcation analysis.

Regardless of these limitations, the importance of the underlying contact network characteristics on the epidemiological outcomes of disease spread is evident. Anecdotally, different types of equine facilities are managed differently. For example, contact between horses in a race facility is often minimized in order to reduce the potential for injury. In contrast, the majority of horses boarded at one of the participating facilities were housed in the same pasture. These differences are likely to result in a range of contact networks with different network densities and degree distributions. The resulting effect of these differences on the disease dynamics coupled with the difference in the disease dynamics between the weighted and unweighted networks, and the consistent difference in disease dynamics between empirical and theoretical networks underlines the importance of using empirical networks to describe the population-level mixing patterns for populations such as the equine population, which are unlikely to exhibit homogenous or random mixing.

## Methods

This study was approved by and conducted in accordance with the University of Guelph’s Research Ethics Board (REB #16AP009) and the Animal Care Committee (AUP #3518). Informed consent was obtained from all study participants prior to the study deployment.

### Empirical networks

Modified OpenBeacon RFID tags (Bitmanufactory ltd., Cambridge, United Kingdom) were used to collect contact pattern data from 4 equine facilities in southwestern Ontario, Canada, over a 7-day period between May and June 2017. Each participating facility contained 1 barn in which the horses’ stalls were located, multiple pastures, and both indoor and outdoor riding areas. The facilities all boarded between 20 and 28 horses (Table 2). Each participating horse wore an RFID tag on their halter for the duration of the study period in order to track and record the number of contacts and duration of each contact that they had with the other horses at the facility. The tags were calibrated to record a contact when the horses were within 2 meters of one another and in the face-to-face position (which was considered a proxy for “close” contact sufficient to transmit a respiratory pathogen). At the end of the 7-day study period, the tags were collected and the resulting contact data were downloaded to a laptop computer where they were stored in a MySQL (Oracle Corporation, Redwood Shores, California) database. The data were aggregated into 24-hour periods and output as comma separated value (CSV) files which were imported into R version 3.3.0^45^ for analysis.

**Table 2:** Demographic information for the participating equestrian facilities. The facilities ranged in size, and discipline. Vaccination rates for equine influenza ranged from 46–100%.

	Facility 1	Facility 2	Facility 3	Facility 4
Number of horses boarded at the facility	20	28	27	24
Number of participating horses	20	28	26*	24
Study date	May-17	May-June 2017	Jun-17	Jul-17
Facility discipline	Dressage	Dressage	Dressage	Therapeutic riding
	Eventing	Jumping		
	Jumping			
Proportion of horses vaccinated for equine influenza (%)	85	100	46.2	100

^*^Although all horses were enrolled in the study, one horse left the facility for the duration of the study, and therefore was not included in the study.

Seven contact networks (1 for each day of the study) were generated for each facility. Each of the 7 contact networks formed a static representation of the contact structure for the respective study day. The 7 daily, static networks were combined to form a dynamic network spanning 7 days, hereafter referred to as *Empirical Networks 1–4*. Both weighted and unweighted networks were produced. In the weighted networks, the edge weight represented the total contact duration between the respective horses on the study day of interest. All of the contact networks were undirected and were composed of multiple components that varied over time. The dynamic empirical networks were composed of 20, 28, 26, and 24 nodes respectively. The normalized degree for each dynamic network ranged between 0.00 and 0.79 (mean = 0.34), 0.00–0.96 (mean = 0.33), 0.00–0.68 (mean = 0.24), and 0.00–0.83 (mean = 0.33). The networks had 46.00–76.00 (mean = 64.29), 33.00–236.00 (mean = 125.43), 28.00–124.00 (mean = 78.00), and 58.00–130.00 (mean = 91.00) edges respectively. A complete description and comparison of each empirical network can be found in^46^.

The Statnet^47^ suite of packages, in particular, the NetworkDynamic^48^ and EpiModel^49^ packages were used for the data preparation, model building, and simulation phases of the study. All data and model code is available in the  Equine non-homogenous mixing GitHub repository.

### Contact network structure

**Figure 4:**
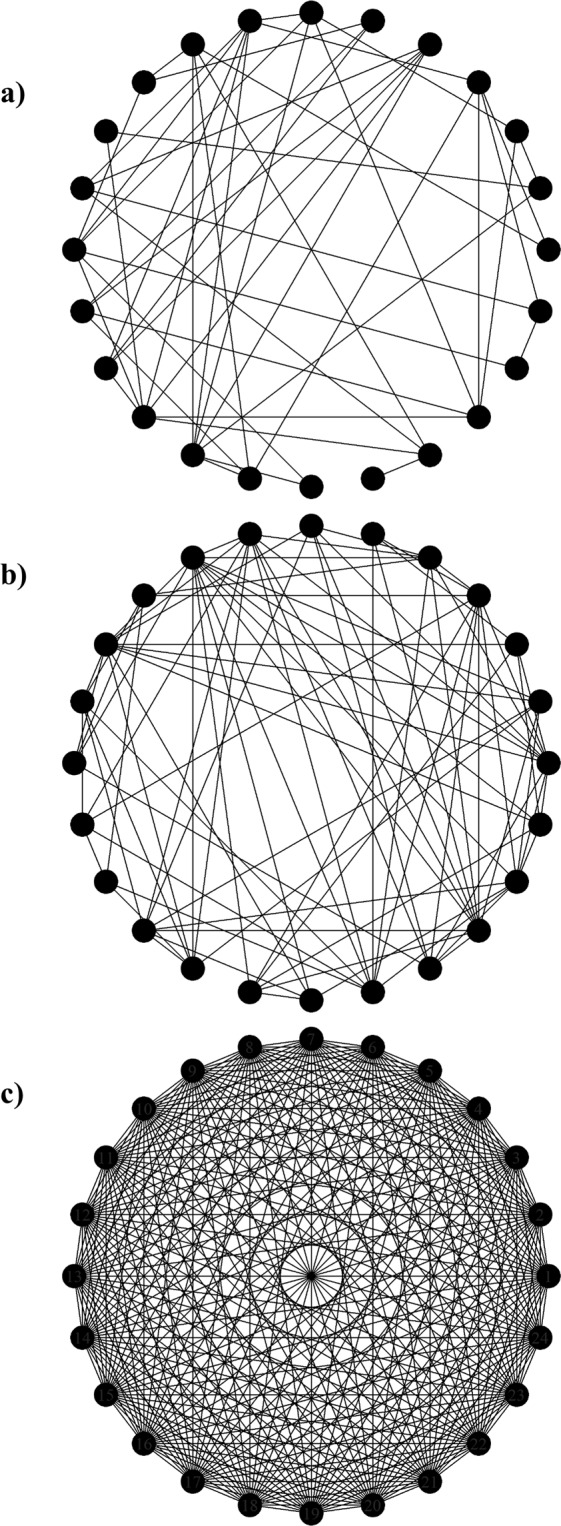
Sample theoretical networks used in the network epidemic model. Panel (a) represents the Minimal Network which has an average of 48 edges. Panel (b) represents the Random Mixing Network which has an average of 87.6 edges. Panel (c) represents a Complete Network in which each node is connected to every other node.

Four types of contact networks were used to quantify the effect of different network structures on the disease transmission model outcomes. The following networks were used: (1) empirical networks (as described above), hereafter referred to as *Empirical Networks 1–4*, (2) a *Random Mixing Network* (*RMN*), (3) a network with a small average degree, hereafter referred to as a “*Minimal Network*”, and (4) a *Complete Network* (Fig. 4). The edge weights for each of the weighted theoretical networks were assigned from a truncated normal distribution such that the weights were all greater than or equal to 0. The distribution was generated using the average network edge weight of each day for each facility (mean = 28.35 hours), and the standard deviation for each day of the study for each facility (standard deviation = 33.35). In addition, the RMN was generated by averaging the characteristics of the empirical networks, namely, the network size, defined as the number of nodes in the network^9^, and the average number of edges in the network. The network size for the RMN was 24. The expected number of edges $({N}_{edges}=87.6)$ was calculated using the mean degree, representing the number of edges incident to a node of interest^9^, of the empirical networks. The Minimal Network was formed by assuming that horses came in contact with an average of 4 other horses per day: the horses whose stalls were on either side of the horse of interest, an average of one horse in the centre aisle and an average of 1 horse in a common riding area. The Minimal Network had an average of 48 edges and is representative of facilities in which horses have reduced contact in order to avoid the potential for injuries^50^. Lastly, the Complete Network was generated using the average network size of the empirical networks (n = 24) and ensuring that all nodes came in contact with every other node in the network. Therefore, the Complete Network had 276 edges $(\frac{n(n-1)}{2})$, where n represents the number of nodes in the network.

Two types of analyses were completed: (1) the effect of weighted vs. non-weighted networks on the disease dynamics was examined, and (2) the disease dynamics were compared for both empirical and theoretical, random mixing networks. For both of these objectives, the 7 day-long static networks were combined to create 1 week-long dynamic network (each daily network was used to inform the contact rate for a single day of the model simulation). The model simulations, however, were run for a 6-week period, creating the need for additional contact pattern data. Therefore, given the repetitive nature of the equine schedule, the empirical week-long dynamic contact networks were repeated to form a 6-week dynamic network. A similar process was used to generate the contact structure for the theoretical networks. Specifically, 7 random networks were created using the methods described above. The 7 networks were combined to form a week-long network, which was repeated for a 6-week period.

### Equine influenza disease transmission model

**Figure 5:**
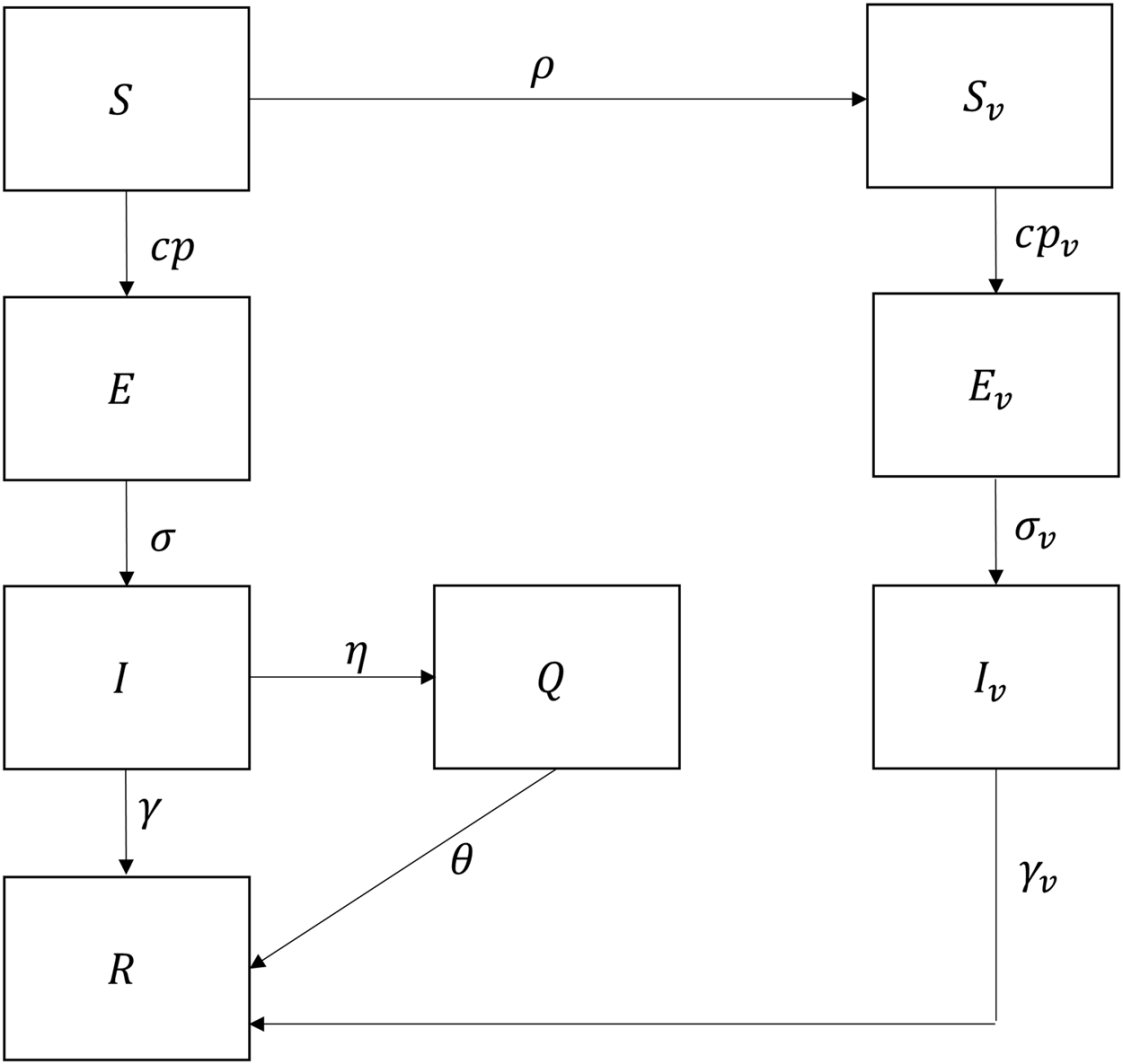
Deterministic analogue of the study model. In this model, horses start out susceptible to infection (S). A proportion ($\rho$) of the susceptible horses are vaccinated (S_*v*_). Both vaccinated and unvaccinated horses can come in contact. Furthermore, both vaccinated and unvaccinated horses can become infected (E), however the rate of infection differs between the vaccinated horses (transmission probability = ${p}_{v}$) and unvaccinated horses (transmission probability = $p$). The transmission rate of the pathogen between infectious horses (I) and susceptible horses is determined through the contact rate (c) and the transmission probability. At the end of the infectious period ($\sigma$ and ${\sigma }_{v}$ for vaccinated and unvaccinated horses respectively), horses can recover (R), where it is assumed that they have full immunity to re-infection. A proportion ($\eta$) of the unvaccinated, infectious horses (I) are isolated (Q). These horses remain in isolation for a pre-determined length of time ($\theta$), before they move to the recovered compartment.

The discrete time, stochastic disease transmission model incorporated a typical SEIR structure and included both vaccination and isolation intervention strategies. A deterministic analogue of the model can be found in Fig. 5. Model parameters were obtained from the peer-reviewed literature (Table 3). The following assumptions were used in the model formation:

1. Horses had no pre-existing immunity to EI.
2. Vaccinated horses were considered to be immune to infection by the start of the simulation.
3. The implications of an imperfect vaccine are that vaccinated horses have a reduced probability of becoming infected, a reduced infectious period, and a longer latent period than unvaccinated horses^32,51^. These implications meant that vaccinated horses could both shed the virus and become infected with the virus^25^. Therefore, the transmission rate, *β*, the latent period, $\sigma$, and the recovery rate, $\gamma \,\,$satisfied the following conditions: ${\beta }_{v} < \beta$, $\sigma  < {\sigma }_{v}$, and ${\gamma }_{v} < \gamma$.
4. The probability of transmission ($p$) was reduced by 50% if at least one of the participating horses in the contact event (either a susceptible horse or an infectious horse) was vaccinated^29,32,51^.
5. The model did not include waning immunity since waning immunity is not expected to occur until approximately 6 months after vaccination and the model was only run for 1.5 months^27,52^.
6. The isolation period was always longer than the infectious period. Therefore, at the end of the isolation period, all horses were assumed to be fully recovered (i.e. horses could no longer shed the virus).
7. Infectious, vaccinated horses were assumed to be asymptomatic, and therefore were not isolated^53^.
8. Given the projected impact of the weighted and non-weighted networks, different transmission probabilities were implemented for each type of network. The transmission probability was calculated as a function of the average edge weights, such that $\beta =\bar{k}\tau$, where $\beta \,\,$is the transmission rate, $\bar{k}$ is the average weighted degree, and $\tau$ is the probability of pathogen transmission. A transmission rate, $\beta ,\,\,$of 1.85 (19) was used to calculate the transmission probability. Therefore, ${\tau }_{w}$ was calculated to be 0.06 for the weighted networks. In contrast, the transmission probability, ${\tau }_{u}$, for the models informed with the unweighted networks, was assumed to be 100% (19), since the transmission probability cannot exceed 100% and $\beta =1.85$.

**Table 3:** List of parameters and variables used in the network epidemic model. Parameter values were obtained from the peer-reviewed literature.

Parameter/variable	Interpretation	Value	Citation
${\boldsymbol{\rho }}$	Proportion of the population that was vaccinated	0–1	Assumed
${{\boldsymbol{p}}}_{{\boldsymbol{v}}}$	Infection probability for vaccinated horses	$0.5\ast \tau$	^32, 51^
$p\,({{\boldsymbol{\tau }}}_{{\boldsymbol{w}}},{{\boldsymbol{\tau }}}_{{\boldsymbol{u}}})$	Infection probability for unvaccinated horses	0.06,1	^32^
$c\,$	Number of acts per encounter	1	Assumed a binary network
${{\boldsymbol{\sigma }}}_{{\boldsymbol{v}}}$	Latent period for vaccinated horses	$\frac{1}{2.52}\,(day{s}^{-1})$	^32, 51^
${\boldsymbol{\sigma }}$	Latent period for unvaccinated horses	$\frac{1}{1.75}$ $(day{s}^{-1})$	^32, 51^
${{\boldsymbol{\gamma }}}_{{\boldsymbol{v}}}$	Recovery rate for vaccinated horses	$\frac{1}{2.5}$ $(day{s}^{-1})$	^32, 51^
${\boldsymbol{\gamma }}$	Recovery rate for unvaccinated horses	$\frac{1}{4.8}\,(day{s}^{-1})$	^32, 51^
${\boldsymbol{\theta }}$	Duration of isolation	$\frac{1}{14}(day{s}^{-1})$	Assumed
${\boldsymbol{\eta }}$	Proportion of infectious horses isolated	0–1 (%)	Assumed
S	Susceptible population		
E	Exposed population, i.e. horses that are infected but not yet infectious		
I	Infectious population		
R	Recovered population		
Q	Isolation compartment for infectious, symptomatic horses		

The parameter symbol represents parameters found in the deterministic analogue (Fig. 2).

The model incorporated 3 different prevention and control strategies that were compared to the baseline (no-intervention) model. The interventions considered were: (1) vaccination, (2) isolation, or (3) a combination of vaccination and isolation. The effect of vaccination on the epidemic outcome was studied by varying the proportion of horses vaccinated at the start of the simulation. Similarly, the effect of isolation was evaluated by varying the proportion of infectious, symptomatic horses isolated throughout the simulation. The proportion of horses isolated was used as a proxy for different levels of symptom severity. For example, it was assumed that horses with less severe symptoms would be less likely to be identified as infectious, and consequently, were less likely to be isolated. Horses were isolated for 14 days and were assumed to be fully recovered after the isolation period.

At the start of the model, a random horse was assigned to the “infectious” status. Additionally, random horses were assigned a status of “vaccinated” according to the specified proportion to vaccinate, ${\rm{\rho }}$. The occurrence of each transmission or transition event was determined using a binomial distribution. The probability of transmission for each contact was calculated as $1-{(1-{\rm{p}})}^{\mathrm{act}\mathrm{rate}}$, where p represents the probability of transmission, and the act rate is the contact frequency. If an infectious, unvaccinated horse came in contact with a susceptible, unvaccinated horse, the infectious horse could transmit the disease to the susceptible horse with a probability *p* (Fig. 5). If either the infectious horse or the susceptible horse was vaccinated, then the probability of transmission, *p*, was reduced by 50%^29,32,51^. Infected horses could progress from the exposed class to the infectious class at the end of the latent period which was 2.52 days for vaccinated horses and 1.75 days for unvaccinated horses.

Once infectious, unvaccinated horses could be isolated. All infectious horses moved to the recovered compartment at the end of the isolation or infectious period respectively. Recovered horses were assumed to be non-infectious with complete immunity to re-infection for the duration of the model time horizon.

The model, which was run over a 6-week period, was simulated 10,000 times using the initial conditions: $(S,\,E,\,I,R)=(N-1,\,0,\,1,\,0)$. For ease of comparison, the model results at each time point were averaged across all the simulations. Models were analyzed with respect to the cumulative infection incidence defined as the sum of the largest integer less than or equal to the simulated incidence at each time point divided by the total population size, and the daily incidence curves.

All R code is freely available on the GitHub repository entitled: Equine non-homogenous mixing.

## Data Availability

All data can be downloaded from the “Equine non-homogenous mixing” GitHub Repository.
